# Comparative Analysis of Short-Chain Fatty Acids and the Immune Barrier in Cecum of Dahe Pigs and Dahe Black Pigs

**DOI:** 10.3390/ani15070920

**Published:** 2025-03-23

**Authors:** Huijin Jia, Yuxiao Xie, Lanlan Yi, Wenjie Cheng, Guangyao Song, Wenzhe Shi, Junhong Zhu, Sumei Zhao

**Affiliations:** 1 College of Animal Science and Technology, Yunnan Agricultural University, Kunming 650201, China; 2 College of Biology and Agriculture, Zunyi Normal University, Zunyi 563006, China

**Keywords:** pigs, cecum, short-chain fatty acids, immune genes, immune barrier

## Abstract

The intestinal immune barrier is a developed and complex immune system, and there is a fine synergy between it and the induced immune response. Short-chain fatty acids (SCFAs) are the main metabolites of intestinal microbial fermentation. In the cecum of pigs, SCFAs not only provide energy for the host but also participate in regulating the function of the intestinal immune system. The purpose of this study was to explore the mechanism of SCFAs in the regulation of immune gene expression in porcine cecum. SCFAs content and mRNA expression levels of immune genes in cecum were detected, and Gene Ontology (GO) function annotation, Kyoto Encyclopedia of Genes and Genomes (KEGG) pathway enrichment analysis, Protein-Protein Interaction Networks (PPI) network construction, key gene identification, and correlation analysis were performed. The results showed that the content of SCFAs in the cecum of Dahe black pigs (DHB) was lower than that of Dahe pigs (DH). There were significant differences in mRNA expression of some immune genes between the two groups. GO functional annotation found terms related to cytokine activity and protein heterodimerization activity; the KEGG pathway was enriched in several pathways related to intestinal immunity. The PPI network identified Interleukin-6 (IL-6), Interleukin-8 (IL-8), Interleukin-10 (IL-10), Interleukin-17A (IL-17A), and Interleukin-18 (IL-18) as key proteins. The correlation analysis showed that acetic acid and valerate were closely related to the immune response. In this study, the differences in cecal short-chain fatty acids and the immune barrier between Dahe pigs and Dahe black pigs were compared, which provided a theoretical basis for improving the intestinal immunity of pigs.

## Simple Summary

The novelty of this study is that the cecal intestinal tissues of Dahe pigs and Dahe Black pigs were studied and the differences in short-chain fatty acids and the immune barrier were compared, which provided basic data for the study of the intestinal health of local pigs species in Yunnan.

## 1. Introduction

In modern pig farming, the healthy growth and efficient breeding of pigs are key to enhancing economic benefits and ensuring a stable supply of livestock products. Maintaining the intestinal health of pigs is a necessary prerequisite. Intestinal homeostasis is a dynamic balance state constructed by the interaction among the intestinal barrier, the intestinal internal environment, and metabolites. In this precise and orderly balance system, the intestinal barrier plays a crucial defensive role. It can effectively resist the invasion of pathogens and maintain the stability of the intestinal internal environment, which is vital to the maintenance of intestinal health.

Short-chain fatty acids (SCFAs) are the primary metabolites produced through the intestinal microbial fermentation of dietary fiber, and they are essential for animal nutrition and intestinal health. In the pigs’ cecum, SCFAs act as an energy source and assist in regulating intestinal immune function. Research indicates that they influence the barrier function of intestinal cells and strengthen defense mechanisms, thereby reducing pathogen invasion [1,2,3,4]. Moreover, SCFAs modulate immune gene expression by engaging G protein-coupled receptors (GPCRs) on intestinal immune cells, influencing immune responses. The activation of these receptors is linked to increased anti-inflammatory responses and immune tolerance [5,6,7]. SCFAs, including acetic, propionic, and butyric acids, are produced by gut microbes as metabolites from the fermentation of undigested carbohydrates. SCFAs, particularly acetic acid, play a crucial role in regulating immune gene expression in the porcine cecum. Acetic acid constitutes 50–70% of the total SCFAs, whereas propionic and butyric acids, which make up 10–25% and 10–30% of SCFAs, respectively, are crucial for intestinal health and immune function [8]. SCFAs influence cell signaling by interacting with intestinal epithelial cells and GPCRs, thus modulating the expression of immune genes [9,10]. For example, butyric acid has been shown to stimulate the proliferation and differentiation of intestinal epithelial cells, produce anti-inflammatory cytokines, suppress inflammatory responses, and is considered the primary energy source for intestinal epithelial cells, playing a particularly crucial role in the immune regulation of the porcine cecum [11]. SCFAs regulate the activity and function of cecal immune cells, influencing intestinal health.

The immune system of the pigs’ cecum is a complex and finely tuned mechanism in which SCFAs play a crucial role. The regulatory mechanisms of SCFAs are essential for overall immune function, exerting diverse and profound effects on the immune process. This includes directly impacting T cell function and regulating the production of pro-inflammatory cytokines by intestinal macrophages and the synthesis of intestinal IgA by B cells. SCFAs are pivotal in maintaining the balance between pro-inflammatory and anti-inflammatory responses [12,13,14,15,16,17,18,19]. Moreover, SCFAs also influence the expression and activity of various immune-related factors across different cell types, thereby enhancing intestinal immune defense capabilities and regulating the activity of immune cells [9,20,21,22,23,24]. As ligands, SCFAs can regulate intestinal immunity by activating receptors such as the aryl hydrocarbon receptor (AhR) [25,26,27,28]. The intestinal immune barrier is a complex local immune system composed of immune organs, cells, and molecules. Intestinal epithelial cells use pattern recognition receptors, especially Toll-like receptors, to distinguish between pathogenic and beneficial substances [12,29,30,31,32,33,34,35,36,37,38,39,40,41,42,43,44]. In summary, the interaction between SCFAs and immune cells, as well as epithelial cells, establishes a complex regulatory network. This network modulates immune gene expression in the pigs’ cecum. The gut immune barrier system and induced immune responses work together to effectively eliminate locally invading pathogens. They also prevent the excessive activation of systemic immune responses, thereby maintaining a balanced and healthy state of the immune system.

The Dahe pig (DH), a native breed from southwest China known for its superior fat-to-meat ratio, is predominantly raised in the Dahe and Yingshang areas of Fuyuan County, Yunnan Province. This breed is known for its early sexual maturity, exceptional mothering skills, strong stress tolerance, ability to thrive on roughage, and meat that is tender and flavorful with a high muscle fat content. The Dahe black pig (DHB), a leaner variety, is celebrated for its superior meat quality, abundant muscle fat, swift growth, exceptional reproductive capabilities, and strong resilience to feeding challenges and stress. The Dahe black pig is particularly well-suited for breeding lean pigs, making it ideal for producing “cloud legs”, both in purebred form and when crossed with Yorkshire hybrids [45].

This study compared the short-chain fatty acid immune DEGs (differentially expressed genes) in the caecal intestinal tissues of Dahe pigs and Dahe black pigs using gas chromatography and real-time fluorescence quantitative PCR. The aim was to explore the mechanism of short-chain fatty acid regulation on caecal immune gene expression, thereby providing basic data for future research on the intestinal health of local pigs in Yunnan.

## 2. Materials and Methods

### 2.1. Experimental Animals and Sample Collection

In this study, both DH and DHB were uniformly raised and managed by the Dahe pigs Breeding Farm (core population farm) in Fuyuan County, Yunnan Province. A total of 180 pigs, consisting of 90 Dahe pigs and 90 Dahe black pigs, weighing 30.00 ± 2.54 kg, were selected. The pigs of each breed were in good condition and were evenly divided between males and females. Each pig was housed in a separate stall and fed the same basic diet, with free access to food and water in identical environments. Composition and Nutrient Levels of Basal Diets are presented in Appendix A. The pig house is maintained with scientifically optimized environmental parameters, including a temperature controlled within 18–26 °C, humidity regulated between 60–80%, and a stocking density complying with industry standards of 1.1–1.3 m^2^/head for finishing pigs. Through comprehensive ventilation systems and spatial layout design, all pens achieve uniform temperature–humidity distribution with variations maintained within ±2 °C and ±5% RH, respectively. This controlled environment ensures thermal comfort and air quality consistency (NH_3_ < 20 mg/m^3^, H_2_S < 10 mg/m^3^) across all animal zones. Upon reaching a weight of 120 ± 6.83 kg, 6 pigs (half male and half female) were randomly selected for slaughter in two groups. Cecal tissues and contents were collected and placed into 1.5 mL frozen storage tubes then stored in liquid nitrogen for subsequent analysis. The research proposal and related experimental procedures have been approved by the Animal Care and Use Committee of Yunnan Agricultural University (Case Number: 20210915).

### 2.2. Test Diet

The dietary formula for this experiment was designed in accordance with the nutritional requirements for local breeds of dual-purpose pigs, intended for both meat and fat production, as specified in the pigs feeding standard using http://down.foodmate.net/standard/sort/3/91362.html (accessed on 10 May 2024). Diets for different weight stages (30–60 kg and 60–120 kg) were prepared. The dietary composition and nutritional levels are detailed in Appendix A.

### 2.3. Detection of SCFAs in Cecal Contents

The precise amounts of acetic acid, propionic acid, butyric acid, and valeric acid were measured, and a mixed standard reserve solution with a concentration of 50 mg/mL was prepared using 50% ethanol. The mixed standard solution was then diluted incrementally to prepare solutions with concentrations of 100, 50, 10, 5, 1, 0.1, and 0.05 μg/mL, respectively. The pre-treated samples and mixed standards were analyzed using gas chromatography–mass spectrometry.

Gas chromatography conditions: Helium is used as the carrier gas, with a controlled flow rate of 1 mL/min. The column temperature was initially set at 70 °C for 0.6 min then increased to 200 °C at a rate of 25 °C/min for 4 min and, subsequently, to 280 °C at 15 °C/min for 5 min. The detector temperature was maintained at 280 °C, the inlet temperature was set at 250 °C, and the injection volume was 1 μL. Mass spectrum conditions: Electron ionization (EI ion source); electron bombardment energy, 70 eV; ion source temperature, 230 °C; MS quadrupole temperature, 150 °C; solvent delay of 4 min. Following the test, a standard curve for SCFAs was plotted based on the concentration and peak area of the standard solution. By substituting the peak area of SCFAs into the linear regression equation, the concentrations of various SCFAs were obtained.

### 2.4. Detection of the Expression Levels of Immune Genes Related to SCFAs

RNA was extracted from porcine cecum tissue using the TRI Reagent protocol, a total RNA extraction reagent from Biosharp (Hefei, China). The qualified total RNA extracted from the cecal tissue was reverse-transcribed into cDNA with the PrimeScript^TM^ RT reagent Kit with gDNA Eraser from TaKaRa (Beijing, China). The mRNA sequences of the genes used in this experiment were sourced from NCBI, and the 18S rRNA gene served as the reference gene. The primers necessary for the experiment were designed using Primer-blast and subsequently synthesized by Tsingke (Kunming, China). qPCR was conducted using the Tli RNaseH Plus fluorescent quantitative kit from TransGen (Beijing, China) (RR820A), with a total reaction volume of 20 µL. This included 0.4 µL of each PCR Forward Primer and PCR Reverse Primer, 10 µL of 2 × PerfectStart Green qPCR SuperMix (TransGen, Beijing, China), 7.7 µL of Nuclease-free Water, and 1.5 µL of cDNA. The reaction was performed on a qPCR instrument from Bio-Rad (CA, USA), with the following cycling conditions: an initial denaturation step at 94 °C for 3 min, followed by 44 cycles of 94 °C for 5 s, 52.8 °C for 15 s, and 72 °C for 10 s. The relative expression levels of the target genes were calculated using the 2^−ΔΔCt^ method, with glyceraldehyde-3-phosphate dehydrogenase (*GAPDH*) serving as the reference gene for data normalization. The Primer Sequence and Annealing Temperature of the Target Gene and Internal Reference Gene are presented in Appendix B. Each sample was analyzed in triplicate to ensure repeatability, and the results are presented as the mean ± standard deviation (SD).

### 2.5. Functional Annotation of SCFAs Immune Genes

The Omicsmart (website: http://www.omicsmart.com) (accessed on 10 January 2025) was utilized to perform gene ontological (GO) enrichment analysis on differentially expressed genes (DEGs) to ascertain their primary biological functions. Additionally, Kyoto Encyclopedia of Genes and Genomes (KEGG) pathway enrichment analysis was conducted on DEGs to elucidate how short-chain fatty acids influence the immune response and signal transduction pathways in the porcine cecum by modulating the expression of specific immune genes. Enrichment of GO terms and KEGG pathways with an adjusted *p*-value (Benjamini) below 0.05 is deemed significant. The enrichment dot plot and KEGG pathway graph were generated using https://www.bioinformatics.org (accessed on 10 January 2025), an online platform dedicated to data analysis and visualization. The PPI network was planned using https://cn.string-db.org/cgi/input?sessionId=bGWJhmXjxChX&input_page_show_search=on (accessed on 15 January 2025), a database designed for constructing functional protein association networks, with a minimum required interaction score of 0.4.

### 2.6. Statistic Analysis

The experimental data were sorted using Excel 2016, and an unpaired *t*-test analysis was conducted on the cecal short-chain fatty acid content of the Dahe pigs and Dahe black pigs, as well as the results of the qPCR test, using SPSS 22.0. The threshold of significance was set at *p* < 0.05. Data normality was verified by the Shapiro–Wilk test (α = 0.05). The homogeneity of variance was tested by Levene’s test. All datasets were tested for normality and homogeneity of variance (*p* > 0.05), which met the premise of parameter testing. All the data satisfied the parameter test hypothesis, so the non-parametric method was not used. Data that fit the parameter hypothesis were compared between groups using student *t* tests. The data were expressed as means ± SEM, with *p* < 0.05 indicating a significant difference and *p* < 0.01 indicating an extremely significant difference. Each gene expression was repeated three times, and the histogram of expression results was drawn using Graph Pad Prism 9.0. Spearman correlation analysis was performed with the assistance of the Sichuan biological cloud platform (https://www.omicstudio.cn/tool, accessed on 10 January 2025), utilizing the correlation clustering tags heat map tool to draw the correlation matrix.

## 3. Results

### 3.1. Content of Short Chain Fatty Acids in Cecum Tissue

**Figure 1:**
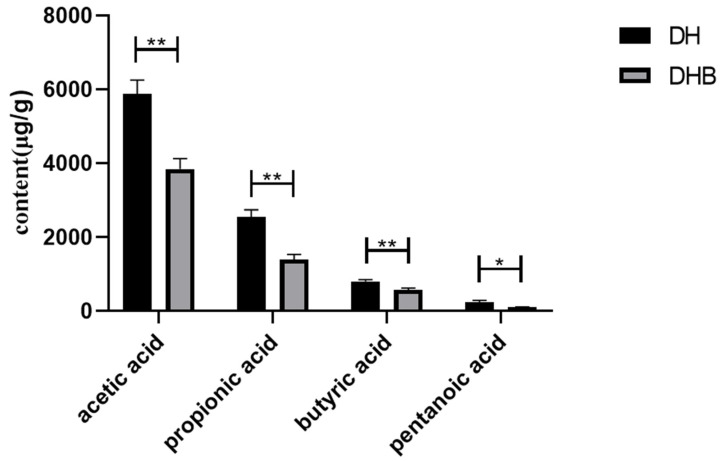
Comparison of short-chain fatty acids in cecum between Dahe pigs and Dahe Black pigs. * indicates significant difference between groups (*p* < 0.05) and ** indicates difference is extremely significant between groups (*p* < 0.01).

Compared with the DH group, the content of pentanoic acid in the cecal tissue of the DHB group was significantly lower (*p* < 0.05), and the levels of acetic acid, propionic acid, and butyric acid were also significantly reduced compared to the DH group (*p* < 0.01), as illustrated in Figure 1.

### 3.2. The mRNA Expression Levels of Immune Genes Associated with SCFAs in Cecum Tissue

**Figure 2:**
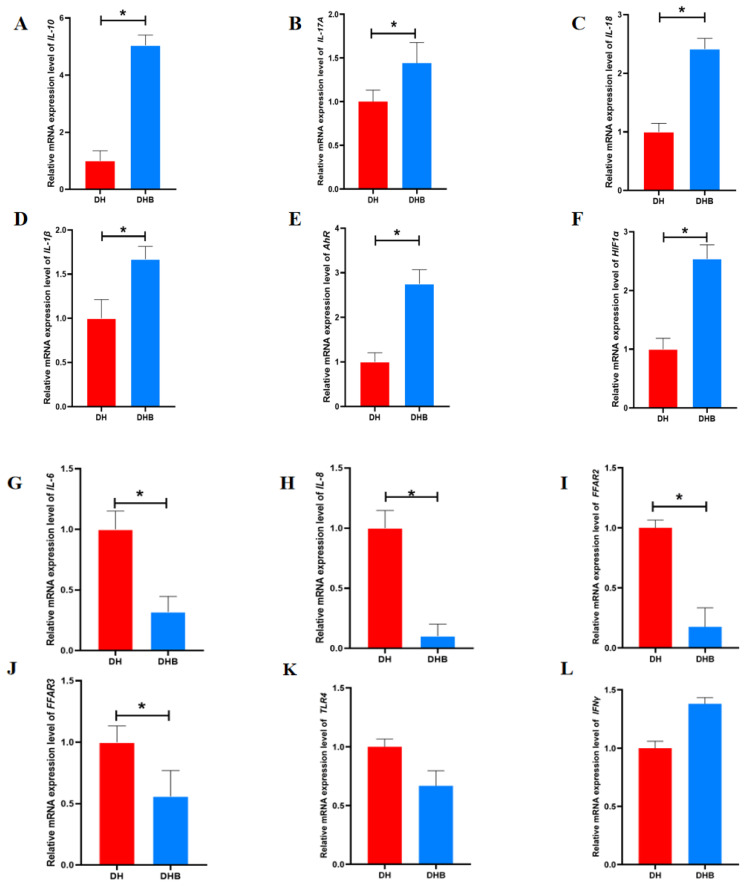

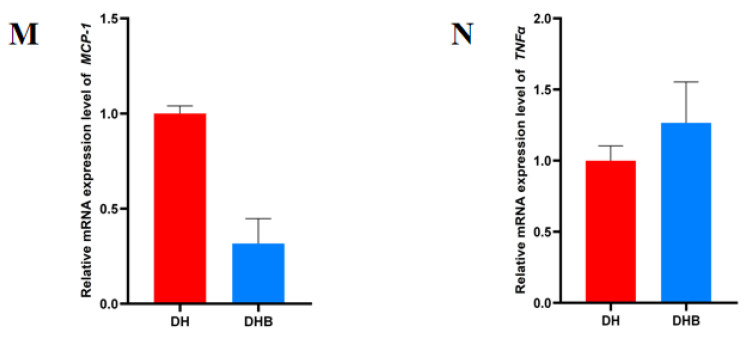
Relative expression of cecum short-chain fatty acid immunity gene mRNA in Dahe pigs and Dahe black pigs. The relative mRNA expression levels of (**A**) IL-6, (**B**) CXCL8 (IL-8), (**C**) FFAR2, (**D**) FFAR3, (**E**) IL-10, (**F**) IL-17A, (**G**) IL-18, (**H**) IL-1β, (**I**) AhR, (**J**) HIF1a, (**K**) TLR4, (**L**) IFN-γ, (**M**) MCP-1, and (**N**) TNF-α. Data are presented as mean ± SEM (n = 6). * indicates significant difference between groups (*p* < 0.05) and ns indicates no statistical difference. *GAPDH* was used as the internal reference gene, and the relative expression was calculated by the 2^−ΔΔCt^ method.

The mRNA expressions of *Interleukin-10* (*IL-10*), *Interleukin-17A* (*IL-17A*), *Interleukin-18* (*IL-18*), *Interleukin-1β* (*IL-1β*), *AhR*, and *Hypoxia inducible factor-1α (HIF1α)* genes in DHB group were significantly higher than those in DH group (*p* < 0.05), while the mRNA expressions of *Interleukin-6* (*IL-6*), *Interleukin-8* (*IL-8*), *Free fatty acid receptor 2 (FFAR2)*, and *Free fatty acid receptor 3 (FFAR3)* genes in DHB group were significantly lower than those in DH group (*p* < 0.05). There were no significant differences in mRNA expression levels of *Toll-like receptor 4 (TLR4)*, *Interferon-γ (IFN-γ)*, *Monocyte Chemoattractant Protein-1* (*MCP-1*), and *Tumor Necrosis Factor-α* (*TNF-α*) genes between the two groups (*p* > 0.05), as shown in Figure 2.

### 3.3. Go Functional Annotation of Short-Chain Fatty Acid Immune Genes in Cecum Tissue

**Figure 3:**
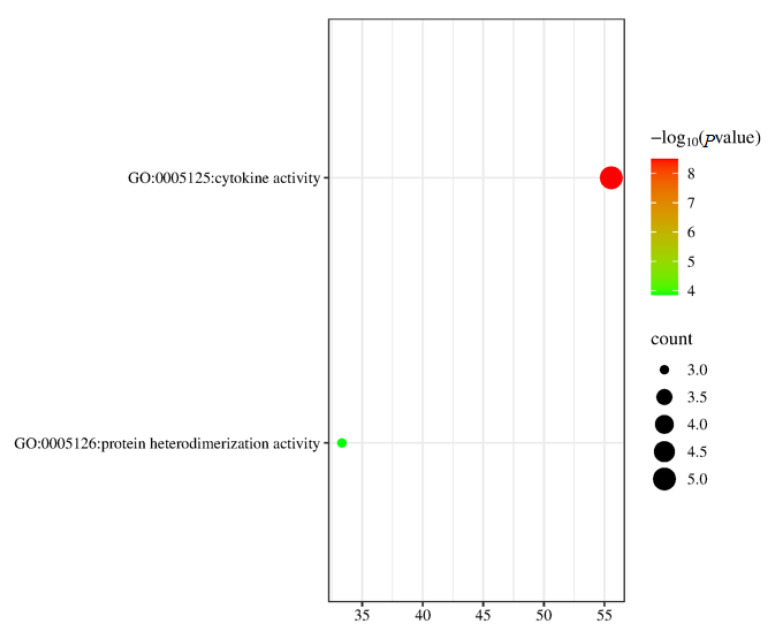
Significantly enriched GO for differentially expressed genes. DH vs. DHB. DH, Dahe pigs; DHB, Dahe black pigs.

Among the 10 DEGs identified between DH and DHB, 8 have been assigned corresponding biological information and functional explanations. There are a total of two terms in this classification of molecular function (*p* < 0.05). These two terms consist of two molecular function items (Figure 3). There is one term related to cytokine activity (GO:0005125) and one term related to protein heterodimerization activity (GO:0046982) (Table 1).

### 3.4. KEGG Functional Annotation of Short-Chain Fatty Acid Immune Genes in Cecum Tissue

**Figure 4:**
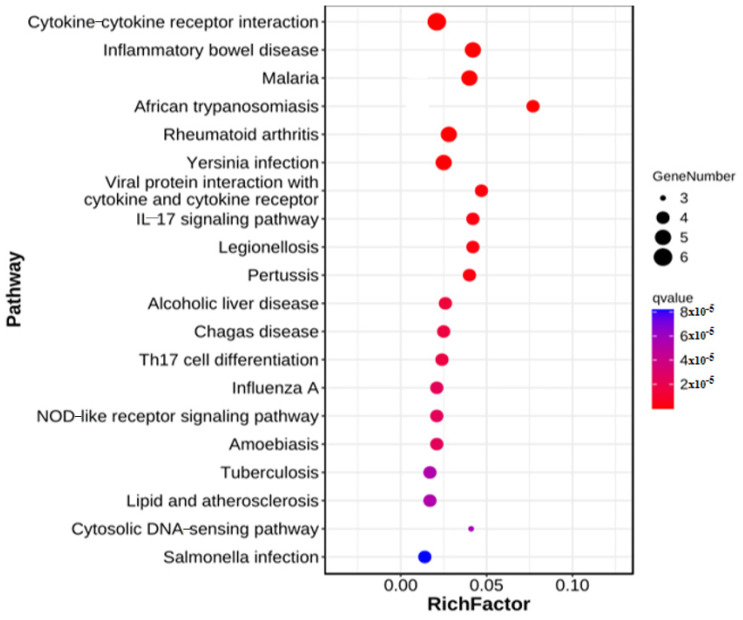
Significantly enriched KEGG for differentially expressed genes. DH vs. DHB. DH, Dahe pigs; DHB, Dahe black pigs.

A total of 10 DEGs between DH and DHB were integrated into the KEGG pathway database, of which 45 pathways (*p* < 0.05) were found to be significantly enriched (Figure 4). Intestinal immunity involves three pathways (Table 2), including inflammatory bowel disease, malaria, Yersinia infection, and the interleukin-17 signaling pathway.

### 3.5. PPI Network Construction and Hub Gene Identification

**Figure 5:**
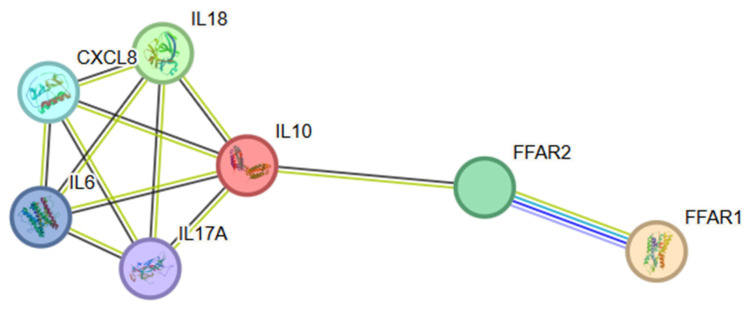
Significantly enriched PPI for differentially expressed genes. DH vs. DHB. DH, Dahe pigs; DHB, Dahe black pigs. Nodes represent proteins, lines represent protein–protein associations, green lines indicate text mining, black lines indicate co-expression, blue lines come from curated databases, purple lines indicate gene co-occurrence, and gray lines represent protein homology.

Protein–protein interaction (PPI) network analysis was performed on key genes in screened DEGs to better understand their interactions. In the DHB-DHB PPI network, 7 nodes and 12 edges are established (Figure 5). We found four distinct clusters in the network, made up of proteins encoded by DEGs. Most proteins in the network interact with multiple different partners. However, we found that interleukin-6 (IL-6), Interleukin-8 (CXCL8), Interleukin-10 (IL-10), Interleukin-17A (IL-17A), and Interleukin-18 (IL-18) had the highest degree of connectivity (four edges). This may enable them to function as “hub” proteins, acting as controllers in biochemical pathways.

### 3.6. Correlation Analysis

**Figure 6:**
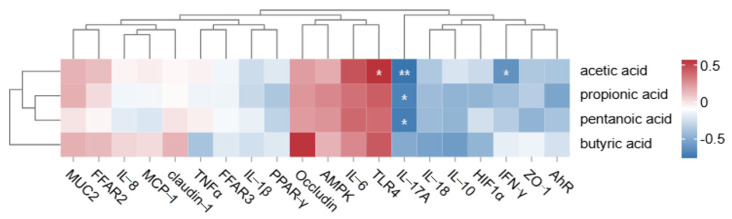
Heat map of short-chain fatty acid content in cecum and expression of genes related to metabolism between Dahe pigs and Dahe black pigs. * means *p* < 0.05, ** means *p* < 0.01.

Based on the content of short-chain fatty acids in the cecum of Dahe pigs and Dahe black pigs and the expression levels of genes related to metabolism, the Spearman correlation coefficient was calculated, and the correlation heat map was constructed, as shown in Figure 6. *TLR4* was positively correlated with acetic acid (*p* < 0.05), *IFN-γ* was negatively correlated with acetic acid, *IL-17A* was negatively correlated with acetic acid, and *IL-17A* was negatively correlated with valeric acid (*p* < 0.05).

## 4. Discussion

As a key product of intestinal microbial metabolism, SCFAs not only provide energy for intestinal flora and host intestinal epithelial cells but also help maintain intestinal pH balance and inhibit the growth of harmful microorganisms. Additionally, SCFAs play a crucial role in regulating the host intestinal immune system, exhibiting immunomodulatory properties and aiding in the reduction of inflammatory responses [46,47]. Studies have indicated that acetic acid is metabolized via the Wood-Ljungdahl pathway and the acetyl-CoA formation pathway of pyruvate oxidative decarboxylation [48,49]. Additionally, intestinal flora plays a role in the regulation of propionic acid production through the succinic acid pathway, which is mediated by Bacteroidetes, and the lactic acid pathway, which is mediated by Firmicutes [50]. As the third major SCFAs, butyric acid is formed by the condensation of two molecules of acetyl Coenzyme A and subsequent reduction to butyryl Coenzyme A. This can be converted to butyric acid by specific microorganisms through the classical pathway involving phosphotransferase and butyric kinase. Additionally, butyric acid can also be produced via an alternative pathway that utilizes exogenous acetate [51]. SCFAs are involved in various cell types, regulating key biological processes such as host metabolism, intestinal function, and immune response. As the shortest carbon chain, formic acid regulate the REDOX balance by participating in glucose metabolism, modulating yeast enzyme activity, and offering anti-oxidative properties. As a major SCFA, acetic acid can participate in cellular metabolic pathways, maintain intestinal integrity, and regulate lipid and carbohydrate metabolism [1]. Propionic acid has been found to possess anti-inflammatory and antibacterial properties, which aid in protecting the gut from pathogens. Additionally, propionic acid can also decrease liver cholesterol synthesis and enhance lipid metabolism [52]. As an energy source for colon cells, butyric acid regulates various intestinal functions, including immune regulation, intestinal development, cell differentiation, and gene expression, thereby reducing oxidative stress and inflammation [53]. Despite the relatively scant research focus on valeric acid and caproic acid, studies have indicated that they possess positive health benefits, including the inhibition of the growth and differentiation of liver cancer cells and the prevention of radiation damage [54,55,56]. In addition, SCFAs also play a role in the prevention and treatment of various diseases, such as obesity [57], cardiovascular diseases [58], diabetes [59], neurological diseases [60], and periodontal diseases [61]. The findings emphasize the importance of SCFAs in regulating intestinal barrier function and overall health. However, there has been limited focus on strains with metabolic properties related to formic acid, valerate, and caproic acid, with only megalococcus reported to produce valerate. It is expected that future research will identify more bacteria capable of producing formic acid and valeric acid, thus clarifying their health implications. This study revealed that the levels of caecal acetic acid, propionic acid, butyric acid, and valeric acid in Dahe black pigs were lower than those in another breed, and the content of caecal valeric acid between the two breeds was significantly different. In summary, it is speculated that Dahe pigs have a stronger ability to regulate key biological processes, such as host metabolism, intestinal function, and immune responses, compared to Dahe black pigs.

Identifying key candidate genes involved in short-chain fatty acid immunity is effective for studying the molecular genetic regulation mechanism of porcine cecal immunity. Within the immune system, the receptor *FFAR2* for acetate, propionate, and butyrate is expressed on eosinophils, basophils, neutrophils, monocytes, dendritic cells, and mucosal mast cells [62,63,64,65]. This indicates that acetate, propionate, and butyrate play a diverse array of roles in the immune response. *FFAR2* diminishes the synthesis of pro-inflammatory cytokines *IL-1β* and *IL-6* by inhibiting the activation of the pro-inflammatory transcription factor, nuclear factor kappa-B (NF-κB) [66]. Upon binding with SCFAs, *FFAR3* primarily functions in bone marrow synthesis by promoting the differentiation of immune cells, thereby regulating the body’s immune response [67]. Human peripheral blood mononuclear cells were treated with SCFAs concentrations of 2 μmol/L, 20 μmol/L, and 200 μmol/L, respectively. It was found that SCFAs down-regulated the expression of host pro-inflammatory factors *IL-1β*, *IL-6*, and *TNF-α*, primarily through the *TLR4* pathway [68]. In this study, the mRNA expression levels of *FFAR2*, FFAR3, and *TLR4* genes in the DHB group were found to be lower than those in the DH group, whereas the mRNA expression levels of the *IL-1β* gene were higher. In conclusion, Dahe pigs may possess a stronger immune regulatory ability in the cecal intestine.

In this study, we concentrated on analyzing differentially expressed genes (DEGs) between two distinct breeds of pigs, DH and DHB. Through an in-depth examination of these 10 DEGs, we successfully annotated eight of them, providing crucial insights into their underlying biological functions. Specifically, at the molecular function classification level in gene ontology (GO), we identified two terms that were significantly significant based on stringent statistical thresholds (*p* < 0.05). These two terms are further refined into two specific molecular functional terms, which reveal important aspects of the differences between the molecular mechanisms of DH and DHB. A term related to cytokine activity (GO:0005125) suggests that cytokine-mediated signaling pathways may play a significant role in both DH and DHB pig species. Cytokines, as the key mediators of intercellular communication, are highly likely to affect a series of biological processes, including cell proliferation, differentiation, and immune regulation, thereby maintaining their differences [69]. This discovery encourages further studies to focus on the upstream and downstream regulatory molecules associated with cytokines to explore potential regulatory networks. Another term related to protein heterodimerization activity (GO:0046982) also warrants attention. Protein dimerization, particularly heterodimerization, often imparts unique structural and functional properties to protein complexes [70]. In the DH and DHB groups, the differential expression of genes involved in this activity suggests that the assembly or dissociation dynamics of specific protein heterodimers differ between the two groups. This change may regulate key cellular processes, such as enzyme activity and transcription factor function, and could be another core factor affecting the biological phenotype of the two states. Collectively, these two terms that distinctly differentiate the DH and DHB groups in terms of molecular function offer direct evidence for elucidating the inherent molecular distinctions between the two groups and indicate the trajectory for future research. On the one hand, we can verify the function of related genes in cellular and animal models through experimental means and further confirm the causal relationship between these genes and the phenotypes of DH and DHB. On the other hand, building upon these two key functional nodes, we can expand our analysis to include other genes, proteins, and metabolites that interact with them. This is expected to construct a comprehensive picture of the molecular regulation of DH and DHB groups, facilitate a deeper understanding of the complex mechanisms underlying related biological phenomena, and lay a solid foundation for theoretical development and potential clinical applications in this field.

In this study, we integrated 10 DEGs between DH and DHB into the KEGG pathway database for analysis, identifying 45 significantly enriched pathways (*p* < 0.05). This provides a wealth of information for understanding the biological differences between DH and DHB. Among these, pathways related to intestinal immunity are of particular interest, including inflammatory bowel disease, malaria, Yersinia infection, and the *IL-17* signaling pathway. The enrichment of these pathways suggests that DH and DHB may have significant differences in intestinal immune regulation. The enrichment of inflammatory bowel disease pathways indicates that molecular mechanism changes may be associated with chronic nonspecific inflammation of the gut in the DH and DHB groups. This could involve an imbalance in the intestinal mucosal immune system, including the activation of immune cells, secretion of cytokines, and alterations in the intestinal microbial community [71]. For example, certain DEGs may play a role in regulating the barrier function of intestinal epithelial cells. Variations in their expression levels could impact the intestine’s resistance to pathogens and immune tolerance towards symbiotic bacteria, which, in turn, is associated with the onset, progression, or disease state of inflammatory bowel disease [72]. The significant enrichment of the malaria pathway suggests that the difference between DH and DHB may be related to the body’s immune response to plasmodium infection. Although malaria is an infectious disease caused by plasmodium parasites, the genetic background and immune status of the host play a key role in susceptibility to infection, disease progression, and outcome [73]. Related DEGs may be involved in the regulation of host cells’ recognition of malaria parasites, the recruitment and activation of immune cells, and the generation of antimalarial immune responses [74]. This finding may provide new insights into studying the immunomodulatory mechanisms of the host during malaria infection and the impact of DH and DHB status on malaria susceptibility. The emergence of Yersinia infection pathways suggests that there may be distinct immune defense mechanisms against Yersinia in the DH and DHB groups. Yersinia includes Yersinia pestis, Yersinia pseudotuberculosis, and Yersinia enterocolitica, which can cause a range of diseases [75]. The enrichment of DEGs within this pathway may be associated with the host cells’ response to the adhesion and invasion of Yersinia, as well as with the immune cells’ process of eliminating the bacteria. For example, certain genes could influence the expression of receptors on the surface of intestinal epithelial cells, thereby altering the interaction between Yersinia and host cells or impacting the production of antimicrobial peptides and cytokines by immune cells, thus affecting the outcome of the infection [76]. The *IL-17* signaling pathway is crucial for intestinal immunity, and its enrichment indicates that DH and DHB differ significantly in this key immunomodulatory pathway. Primarily secreted by Th17 cells, *IL-17* stimulates epithelial cells to produce antimicrobial peptides and chemokines, which, in turn, recruit neutrophils and other immune cells to the site of infection. This pathway is essential for combating extracellular pathogens and maintaining the homeostasis of the intestinal mucosal immune system [77]. The alterations in DEGs within this pathway could impact the production and signaling of IL-17 and the expression of downstream effector molecules, thereby affecting the equilibrium of intestinal immune defense and inflammatory response. In summary, the enrichment of these immune-related pathways highlights the complex regulatory mechanisms and potential disparities in intestinal immune function between DH and DHB. Further studies are required to explore the specific roles of key DEGs within these pathways and their interactions in regulating intestinal immunity. This will enhance our understanding of the biology of DH and DHB and offer new targets and insights for the prevention, diagnosis, and treatment of related diseases. Simultaneously, by integrating other omics data and functional experiments, it is anticipated that a more comprehensive DH and DHB intestinal immune regulatory network will be constructed, offering deeper theoretical support for research in related fields.

In this study, protein–protein interaction (PPI) network analysis has become a key component in the process of in-depth exploration of the molecular mechanism differences between DH and DHB, offering a unique perspective for analyzing the internal associations of selected DEGs. By constructing the PPI network, we have uncovered a series of valuable insights that further deepen our understanding of this biological system. In the obtained DH and DHB PPI network, although only 7 nodes and 12 edges were established (Figure 5), the scale may seem limited, its complexity and functionality are significant and should not be underestimated. It is important to note that the network contains four distinct clusters, and these protein clusters, encoded by DEGs, reflect the initial division of functional modules. This suggests that in DH and DHB, genes and their products do not operate in isolation but work together in accordance with specific functions to coordinate the regulation of related biological processes. Each cluster may represent a relatively autonomous and closely related subsystem, such as a specific module involved in signal transduction, metabolic regulation, or immune response. These subsystems are interwoven to shape the distinct phenotypes of DH and DHB. By examining the connectivity properties of the network more closely, most proteins demonstrate the ability to interact with multiple different partners, emphasizing the dynamic and complex nature of the protein-interaction network and revealing a broad and intricate pattern of collaboration among molecules within the cell. More importantly, IL-6, CXCL8, IL-10, IL-17A, and IL-18 emerge as “hub” proteins within the network, characterized by their connectivity through four edges. The importance of these “hub” proteins lies in their likely role as controllers in the biochemical pathways related to DHB. For example, IL-6, as a key regulator of the inflammatory response, can rapidly activate various signaling pathways during stress states such as infection and trauma. This not only promotes the proliferation and differentiation of immune cells but also regulates the synthesis of acute phase proteins, thereby exerting a broad influence on the immune defense and tissue repair processes of the body [78]. In the PPI network of this study, IL-6 is in a highly connected state, indicating that it may serve as a bridge connecting multiple functional modules. Through interactions with various proteins, IL-6 precisely regulates the entire process from the initiation to the resolution of inflammation and coordinates the differences between DH and DHB in immune stress. Similarly, CXCL8, a potent chemokine, can attract neutrophils and other immune cells to migrate to the site of inflammation, establishing the front line of immune defense at the site [79]. Its close connection with multiple proteins may ensure the efficient and accurate recruitment process of immune cells, ensuring that in DH and DHB, immune cells can respond to different physiological or pathological needs in a timely and accurate manner. IL-10 is known for its powerful anti-inflammatory properties and serves as an important counterbalancing force in maintaining immune homeostasis [80]. Highly connected within the network, it may interact with pro-inflammatory proteins to halt excessive inflammatory responses promptly, preventing immune overreaction that could harm the body, and thus playing a role in maintaining balance. IL-17A plays a crucial role in mucosal immune defense by stimulating epithelial cells to secrete antimicrobial peptides and chemokines, and by cooperating with neutrophils to combat pathogen invasion [81]. Its “hub” position within the PPI network enables it to integrate multiple resources, mobilize immune and epithelial cells to collaborate in defense, and provide tailored immune protection strategies to DH and DHB against external pathogen invasion. As an immunomodulator, IL-18 is involved in activating various immune cells and initiating the connection between innate and adaptive immunity [82]. Its high connectivity indicates that under the DH and DHB system, it is at the key intersection of immune regulatory information flow. By binding with different proteins, it connects innate immunity and adaptive immunity, ensuring the coherence and efficiency of the immune response. In summary, by analyzing the PPI networks of DH and DHB, especially by identifying these “hub” proteins, we not only gain insight into the complex interaction patterns among gene products but also reveal the potential regulatory core. These findings point out the direction for further research on the molecular regulatory mechanisms of DH and DHB: on the one hand, functional verification experiments can be carried out around “hub” proteins and their interacting partners to accurately analyze their specific mechanisms of action in the transformation process of DH and DHB; on the other hand, combined with other omics data, such as metabolomics, transcriptomics, etc., a more comprehensive multi-omics regulatory network can be constructed to fully explain the biological nature of DH and DHB and provide a solid theoretical basis for the diagnosis and treatment of related diseases.

TLRs activate the innate immune system and are typically expressed in immune cells such as B lymphocytes and monocytes [83]. The *TLR4* mechanism is responsible for recognizing components of the extracellular cell walls of pathogens, such as fungi and bacteria, and plays a role in immune recognition. It presents specific receptor information of pathogens to B lymphocytes, thereby exerting its immune effect. *IFN-γ*, encoded by the interferon gene, is the sole type II interferon and is primarily derived from helper T cells (CD4/8+ T cells), Th1 cells, and natural killer (NK) cells [84]. It has certain antiviral, anti-tumor, and promotion of macrophage activation [85]. *IFN-γ* plays a pivotal role in the body’s immune response, being a product of adaptive immunity. It also influences innate immunity. It not only combats pathogens but also participates in immune cell differentiation, activation, and regulation. Immune regulation is achieved through various mechanisms, including enhancing antigen processing and presentation, increasing leukocyte trafficking, inducing antiviral states, boosting antimicrobial functions, and affecting cell proliferation and apoptosis [86]. *IFN-γ* secreted by Th1 cells can induce the innate immune response in a host infected with Toxoplasma gondii or echinococcosis, thus playing an anti-parasitic role [87]. *IL-17* is a pro-inflammatory cytokine secreted by Th17 cells, and its family includes *IL-17A* through *IL-17F* [88]. *IL-17A* has the highest content and the strongest biological activity in the *IL-17* family, which is commonly referred to as *IL-17A* [89]. High expression of *IL-17A* results in the recruitment of neutrophils and chronic infiltration of immune cells [90]. Simultaneously, *IL-17A* can stimulate the production of cytokines via the *IL-17A* receptor pathway, resulting in autoimmunity and tissue damage. Recent studies have discovered that *IL-17A* can modulate the apoptosis process of various cells [91]. In this study, *TLR4* exhibited a positive correlation with acetic acid, while *IFN-γ* and *IL-17A* both showed negative correlations with acetic acid. Additionally, *IL-17A* was negatively correlated with valeric acid, suggesting that both acetic acid and valeric acid are closely associated with the body’s immune response.

In recent years, the role of non-fatty acid metabolites has gradually been revealed. For instance, indole metabolites can bind to and activate the AhR, inducing the expression of downstream cytokines to maintain gut homeostasis [92]. Indole-3-acetic acid (IAA), a metabolite of tryptophan, can induce the secretion of IL-22 via the AhR signaling pathway, thereby promoting the repair of intestinal epithelial cells [93]. Similarly, the metabolite 2-hydroxy-4-methylvaleric acid (HMP), secreted by Bacillus subtilis, was first reported to enhance intestinal barrier function by activating the GADD45A-Wnt/β-catenin pathway, offering a new mechanism for the therapeutic potential of probiotic-derived metabolites [94]. This mechanism complements the GPCR/HDAC pathway of SCFAs and the AhR pathway of IAA, expanding the molecular network of metabolites that regulate intestinal homeostasis. Thus, the synergistic effect of metabolites may be the focus of future research.

## 5. Conclusions

In summary, upon comparing two distinct pigs breeds (DH and DHB), we identified 10 unique differentially expressed genes (DEGs). Among these, the genes *IL-6*, *CXCL8*, *IL-10*, *IL-17A*, and *IL-18* were pinpointed as key regulators of intestinal immunity. These genes dictate the immune response in pigs. The observed disparities in SCFAs levels between two breeds indicate potential differences in their capacity to regulate biological processes. The findings presented in this paper lay a theoretical foundation for enhancing pigs intestinal immunity in the future.

## Figures and Tables

**Table 1:** Significant enrichment related to cytokine activity and protein heterodimerization activity.

Groups	Term ID	Description	*p* Value	Gene Number
DH vs. DHB	GO:0005125	cytokine activity	3.2359365693 × 10^−9^	5
	GO:0046982	protein heterodimerization activity	0.000141253754462	3

**Table 2:** The significantly enriched pathways associated with gut immunity.

Groups	Pathway ID	Description	*p* Value	Gene Number
DH vs. DHB	ko05321	Inflammatory bowel disease	5.60283 × 10^−9^	5
	ko05144	Malaria	7.48289 × 10^−9^	5
	ko05135	Yersinia infection	8.262792 × 10^−8^	5
	ko04657	IL-17 signaling pathway	3.107567 × 10^−7^	4

## Data Availability

The original contributions presented in this study are included in the article/Appendix. Further inquiries can be directed to the corresponding authors.

## Appendix A. Composition and Nutrient Levels of Basal Diets (Air-Dry Basis)

**Item**	**BW (kg)**	
	**30~60**	**60~120**
Raw material (%)		
Corn	64.21	61.50
Soybean meal	16.11	15.00
Rice bran	10.00	10.00
Wheat bran	7.10	10.00
Stone powder	1.08	2.00
Premix	1.00	1.00
Calcium hydrogen phosphate	0.32	0.00
Salt	0.18	0.50
Total	100	100
Nutrient level		
pigs digestive energy (MJ/kg)	13.81	13.51
Crude protein *	15.17	14.85
Lysine *	0.63	0.55
Calcium *	0.55	0.81
Total Phosphorus *	0.53	0.49
(1) Meat fat type growth and finishing pigs (30~60 kg) premix provides the following: vitamin A, 5000 IU; vitamin D_3_, 180 IU; vitamin E, 15 mg; vitamin K_3_, 0.50 mg; thiamine vitamin B_1_, 1.50 mg; riboflavin vitamin B_2_, 2.50 mg; niacin VPP, 12.00 mg; pantothenic acid, B_5_ 9.00 mg; pyridoxine B_6_, 1.00 mg; biotin VH, 0.08 mg; folic acid, 0.30 mg; vitamin B_12_, 10.00 mg; choline, 350 mg; iron, 148.34 mg; copper, 199.50 mg; manganese, 113.50 mg; zinc, 65.00 mg; iodine, 0.15 mg; selenium, 0.25 mg. Meat fat type growth and finishing pigs (60~100 kg) provides the following: vitamin A, 5000 IU; vitamin D_3_, 160 IU; vitamin E, 15 mg; vitamin K_3_, 0.50 mg; thiamin B_1_, 1.50 mg; riboflavin B_2_, 2.50 mg; niacin Vpp, 9.00 mg; pantothenic acid B_5_, 8.00 mg; pyridoxine B_6_, 1 mg; biotin VH, 0.08 mg per kg of diet; folic acid, 0.30 mg; vitamin B_12_, 6.00 mg; choline, 0.40 g; iron, 258.56 mg; copper, 199.50 mg; manganese, 113.50 mg; zinc, 55.00 mg; iodine, 0.15 mg; selenium, 0.25 mg. (2) * is the measured value.		

## Appendix B. The Primer Sequence and Annealing Temperature of the Target Gene and Internal Reference Gene

**Genes**	**Primer Sequences (5′-3′)**	**Annealing Temperature**
*18S rRNA*	F: GTCCACTGGTGTCTTCACGA R: GCTGACGATCTTGAGGGAGT	57.5 °C
*FFAR2*	F: CGTGTTCATCGTTCAGTA R: GAAGTTCTCATAGCAGGTA	51.5 °C
*FFAR3*	F: TGGAGACCTTACGTGTTG R: CGAGGATGAGAAGTAGTAGAT	57 °C
*IL-1β*	F: CCCAAAAGTTACCCGAAGAGG R: TCTGCTTGAGAGGTGCTGATG	57 °C
*IL-6*	F: GGACGCCTGAAGAAGAT R: TGAACCCAATTGGAAGC	51.5 °C
*CXCL8*	F: ACTGGCTGTTGCCTTCTT R: CAGTTCTCTTCAAAAATATCTG	57 °C
*IL-10*	F: GCTGAAGACCCTCAGGCTGA R: TTGCTCTTGCTTCTCCACT	59 °C
*IL-17A*	F: CAGATTACTCCAAACGCTTCACC R: GCAGCCCACTGTCACCATCACTT	57 °C
*IL-18*	F: GCAGTAACCATCTCTGTGCAGTGTA R: TCATCAATATTATCAGGAGGACTCATTT	59 °C
*TNFα*	F: ACCACGCTCTTCTGCCTACTGC R: TCCCTCGGCTTTGACATTGGCTAC	61 °C
*TLR4*	F: ACCAGACTTTCTTGCAGTGGGTCA R: AATGACGGCCTCGCTTATCTGACA	51.5 °C
*IFN-γ*	F: GGCCATTCAAAGGAGCATGG R: AGTTCACTGATGGCTTTGCG	51.5 °C
*MCP-1*	F: CAGCCCTCCTGTGCCT R: TTCAAGGCTTCGGAGTTT	51.5 °C
*AhR*	F: ACTACCACCCATCTTCACCCG R: CAACACACATCAATGCTTCCC	57 °C
*HIF1α*	F: CATCAGTTGCCACTTCCCCAT R: CAAAACCATCCAAGGCTTTCA	57 °C
